# Synergistic effect of the rifaximin–berberine combination against *Klebsiella pneumoniae*: RfaH targeting supported by MD simulation

**DOI:** 10.17305/bb.2026.13776

**Published:** 2026-01-28

**Authors:** Anam Ashraf, Mohammad Ali Khan, Arunabh Choudhury, Swati Kumari, Bader S Alotaibi, Saba Noor, Mohd Adnan, Md Imtaiyaz Hassan

**Affiliations:** 1 Centre for Interdisciplinary Research in Basic Sciences, Jamia Millia Islamia, New Delhi, India; 2 Department of Biotechnology, School of Chemical and Life Sciences, Jamia Hamdard, New Delhi, India; 3 National Institute of Immunology, New Delhi, India; 4 Department of Clinical Laboratory Sciences, College of Applied Medical Sciences, Shaqra University, Al-Quwayiyah, Riyadh, Saudi Arabia; 5 Department of Biology, College of Science, University of Ha’il, Ha’il, Saudi Arabia

**Keywords:** Synergistic effect, rifaximin-berberine, anti-termination protein RFAH, MD simulation, antibiotic resistance, *K. pnuemoniae.*

## Abstract

The escalating crisis of antimicrobial resistance (AMR) among Gram-negative pathogens, particularly *Klebsiella pneumoniae* (KP), necessitates innovative strategies to enhance the efficacy of existing antibiotics. Synergistic drug combinations present a promising approach to improve therapeutic outcomes and delay the emergence of resistance. This study investigates the synergistic interaction between the natural alkaloid berberine chloride and the repurposed antibiotic rifaximin against KP. Integrated *in vitro* and *in silico* analyses reveal significant bactericidal synergy between the two agents, mediated through concurrent inhibition of the transcriptional anti-termination factor RfaH, a key regulator of virulence and capsule biosynthesis. Molecular docking and dynamics simulations demonstrate that both compounds cooperatively bind to the RfaH pocket, stabilizing an inactive ternary complex without major structural disruption. Functional assays confirm that the combination effectively suppresses RfaH-dependent capsule production at lower concentrations compared to monotherapy. These findings suggest that RfaH is a viable target for combinatorial inhibition and provide a plausible mechanistic foundation for the berberine–rifaximin synergy. This work supports the rational development of dual-targeting anti-virulence strategies to combat multidrug-resistant KP infections.

## Introduction

*Klebsiella pneumoniae* (KP) is a significant opportunistic pathogen belonging to the Enterobacteriaceae family. It is not a uniform entity but comprises distinct and clinically relevant phenotypes. KP is responsible for a wide range of diseases and exhibits a troubling trend of escalating antibiotic resistance [1]. Notable variants include classical multidrug-resistant (MDR) strains, hypervirulent (hvKp) strains associated with severe community-acquired infections, and increasingly common hybrid isolates that combine hypervirulence with multidrug-resistance traits, posing a dual threat to public health [2, 3]. In the World Health Organization’s (WHO) bacterial priority pathogens list for 2024, carbapenem-resistant KP was ranked as the top-priority pathogen, receiving a score of 84%, which places antibiotic-resistant KP strains in the highest quartile of concern [4].

In Europe, KP accounts for over 90,000 infections and approximately 7,000 deaths annually, contributing to 25% of disability-adjusted life years lost to bacterial infections. The clinical management of these infections is significantly complicated by the increasing prevalence of multidrug-resistant strains, particularly those producing extended-spectrum β-lactamases (ESBLs) and carbapenemases. This resistance profile often necessitates reliance on last-line treatments, such as colistin in combination with β-lactamase inhibitors, which are suboptimal due to potential toxicity and variable efficacy [5]. A major obstacle to preventing these infections is the lack of effective and licensed vaccination strategies, despite extensive ongoing research efforts [6]. Infections caused by drug-resistant KP represent a critical public health threat, with the continuous evolution and increasing multidrug resistance of KP posing direct risks to human health [7].

The transcriptional elongation factor RfaH plays a crucial role in promoting the expression of long virulence operons in various bacteria. In KP, RfaH is essential for enhancing capsular polysaccharide (CPS) synthesis, hypermucoviscosity, and overall bacterial fitness during infection [8]. Deletion of the *rfaH* gene significantly attenuates virulence in the highly virulent, hypermucoviscous K1 serotype reference strain NTUH-K2044, which causes liver abscesses and meningitis [9]. Impairment of lipopolysaccharide (LPS) biosynthesis due to *rfaH* deletion disrupts the assembly of outer membrane proteins, consequently reducing membrane permeability [10]. Under conditions of iron restriction, the *rfaH* mutant exhibits diminished growth kinetics, primarily due to impaired CPS production. This observation is supported by the established relationship between CPS biosynthesis and iron availability in hypervirulent KP (hvKp). The disruption of RfaH-regulated CPS synthesis likely hinders physiological adaptations critical for optimal growth [11]. These findings position RfaH as a promising target for the development of anti-infective strategies against KP.

As antibiotic discovery rates continue to decline despite efforts from the pharmaceutical industry, the WHO has called for urgent development of novel therapies. Plant-derived natural products (NPs) present a strategic solution, leveraging their historical use as sources of bioactive agents and ethnopharmacological knowledge [12–14]. *Coptis chinensis* Franch, known as Huanglian in Traditional Chinese Medicine (TCM), is a pharmacopoeial herb that exhibits potent broad-spectrum antibacterial activity against conditions such as dysentery, cholera, leukemia, diabetes, and lung cancer. The principal constituents responsible for these pharmacological effects, particularly the antimicrobial properties, are isoquinoline alkaloids, with berberine as the major bioactive component [15].

Research has demonstrated that berberine possesses strong *in vitro* antimicrobial activity against methicillin-resistant *Staphylococcus aureus* (MRSA), with notably low minimum inhibitory concentration values [16]. Berberine’s antimicrobial efficacy has been established in both traditional and modern medical contexts. Recent studies have modified the berberine core to produce a series of novel Schiff base analogues for the development of antimicrobial agents.

The potential of these berberine-derived compounds has been evaluated, revealing enhanced antimicrobial activity against *Pseudomonas aeruginosa*, *Aspergillus fumigatus*, *Staphylococcus epidermidis*, and *Candida albicans*, as demonstrated by free radical scavenging assays [17]. Building on previous studies that highlight berberine’s significant potential as a novel antimicrobial agent, we further explore the successful repurposing of rifaximin to target RfaH in KP [18, 19].

This study investigates the synergistic potential of berberine and rifaximin against KP. Given the substantial challenges associated with discovering entirely new drugs for multidrug-resistant infections such as KP, exploring synergistic combinations of existing or repurposed compounds represents a promising and time-efficient strategy. We employed integrated *in silico* methods (molecular docking and molecular simulation analysis) alongside *in vitro* techniques (checkerboard assays and time-kill curves) to rigorously evaluate the interaction between berberine and rifaximin, aiming to identify effective combination therapies that could expedite therapeutic development.

## Materials and methods

### Materials

Rifaximin, berberine chloride, and ciprofloxacin were obtained from Sigma-Aldrich (St. Louis, MO, USA). Luria–Bertani broth and Nutrient Agar were sourced from HiMedia Laboratories (Maharashtra, India). p-Iodonitrotetrazolium chloride (INT) was purchased from Merck (Darmstadt, Germany). BIOMOL^®^ Green reagent was procured from Enzo Life Sciences (New York, USA). All other chemicals and solvents were of analytical grade.

### Fluorescence measurements

To assess the binding affinity of RfaH for rifaximin, berberine chloride, and their combination, a fluorescence-quenching study was conducted using a Jasco FP-8200 spectrofluorometer (Japan). Stock solutions of both compounds were prepared in dimethyl sulfoxide (DMSO) at a concentration of 50 mM, then diluted in Tris-NaCl buffer (50 mM Tris-HCl, 150 mM NaCl, pH 7.5) to a final working concentration of 0.5 mM. During the experimental runs, the RfaH concentration was maintained at 8.0 µM.

For individual interaction experiments, rifaximin and berberine chloride were titrated into the RfaH solution at a 1:9 ratio, with additions continuing until no further quenching was observed. In the combination assays, both compounds were pre-mixed in a 50 µM rifaximin to 6.25 µM berberine chloride ratio (8:1 molar ratio), corresponding to the synergistic concentrations identified in microbiological assays. This mixture was then titrated into the RfaH solution using the same titration procedure. Fluorescence emission was recorded over the range of 300–400 nm, with excitation set to 280 nm to target tryptophan residues. Slit widths and sensitivity settings were standardized across all spectral parameters to ensure data consistency. Fluorescence quenching parameters were analyzed using a modified Stern–Volmer relationship to determine the binding constant (*Ka*) and the number of binding sites (*n*) for each test condition: rifaximin alone, berberine chloride alone, and their equimolar mixture. All experiments were conducted in triplicate, and corrections were applied to eliminate background fluorescence from the buffer and potential inner-filter effects. By integrating these steps into the workflow, we accurately characterized the interaction of RfaH with the individual compounds and their combination [20–22].

### Minimum inhibitory concentration measurements

The bacterial strain used was KP ATCC 700603, a widely utilized ESBL-producing reference strain, phylogenetically classified within *Klebsiella quasipneumoniae subsp. similipneumoniae*. For consistency with the ATCC designation and common literature references, we refer to it herein as KP ATCC 700603. To evaluate antibacterial efficacy, the minimum inhibitory concentrations (MICs) of rifaximin, berberine chloride, and their combined formulation were determined against KP (ATCC 700603) using a p-iodonitrotetrazolium chloride (INT)-based colorimetric assay. Each compound was initially dissolved in DMSO, with the solvent concentration carefully limited to below 2.5% in the final mixtures to prevent any adverse effects on bacterial viability. For single-agent testing, serial two-fold dilutions were prepared in broth across the rows of a 96-well microtiter plate, with final concentrations ranging from 200 µM to 0.78 µM. Wells were inoculated with 100 µL of bacterial suspension (∼1.5 × 10^5^ CFU/mL). For the combination study, the checkerboard method was employed as described below.

### Checkerboard assay

To assess potential drug–drug interactions, a checkerboard microdilution method was utilized. Rifaximin was serially diluted two-fold across the rows (final concentration range: 100 µM to 0.78 µM), while berberine chloride was similarly diluted down the columns (final concentration range: 100 µM to 0.78 µM). Each well contained a unique combination of both agents. The assay plate wells were inoculated with 100 µL of bacterial suspension KP (ATCC 700603, approximately 1.5 × 10^5^ CFU/mL) and incubated at 37 ^∘^C for 18 h. Following this incubation, INT dye was added to achieve a final concentration of 0.2 mg/mL, and the plates were incubated for an additional 30 min under the same conditions. A reduction of yellow INT to a pink hue indicated active bacterial metabolism. The MIC for each well was defined as the lowest concentration at which the dye retained its original yellow color, signifying complete inhibition of bacterial growth. Control groups included wells containing only bacterial cultures without drug exposure and wells with sterile medium to assess background signals. Ciprofloxacin-treated wells served as a control for growth inhibition. Each assay was conducted in triplicate to ensure reproducibility. In experiments involving both rifaximin and berberine chloride, the fractional inhibitory concentration index (FICI) was calculated to quantify their interaction and categorize the effects as synergistic, additive, or antagonistic [23].

The FICI was calculated using the equation:

FICI ═ (MIC_A in combination / MIC_A alone) + (MIC_B in combination / MIC_B alone)

Where MIC_A and MIC_B represent the minimum inhibitory concentrations of rifaximin and berberine chloride, respectively, determined either alone or in combination.

### Determination of minimum bactericidal concentration

Time-kill assays were conducted to investigate the bactericidal effects of rifaximin, berberine chloride, and their combined treatment against KP (ATCC 700603). Bacterial cells in the early logarithmic phase were exposed to each agent individually and in combination at concentrations corresponding to their respective MICs. For combination testing, drug concentrations were selected based on the synergistic ratios determined in the prior checkerboard assay. Samples were collected at 0, 3, 6, 9, and 24 h, serially diluted, and plated onto nutrient agar to count viable colonies after 24 h of incubation. Two sets of controls were utilized: untreated bacterial cultures as the growth control and sterile medium to verify sterile conditions. CFU counts were graphed on a logarithmic scale (y-axis) against time (x-axis) to evaluate bactericidal dynamics. All experiments were performed in triplicate to ensure reproducibility. This approach facilitated direct assessment of the time-dependent bactericidal activity of rifaximin, berberine chloride, and their combined treatment against KP [24]. Synergy in time-kill assays was defined as a ≥2 log_10_ CFU/mL decrease in viable counts by the combination compared to the most effective single agent at the same time point [25].

### Capsule quantification assay

CPS synthesis and uronic acid content of KP (ATCC 700603) were evaluated using a capsule quantification method adapted from previously reported methods [26, 27]. Cultures grown in 20 mL LB broth were incubated for 16 h under four conditions: 100 µM rifaximin, 100 µM berberine chloride, a combination of both, or no treatment (control). For combination treatment, cultures were exposed to 50 µM rifaximin and 6.25 µM berberine chloride simultaneously. Bacterial concentrations were standardized based on CFU/mL counts prior to CPS extraction. CPS extraction followed a modified Zwittergent-based method [28], in which 500 µL of culture was combined with 1% Zwittergent in 100 mM citric acid, incubated at 50 ^∘^C for 20 min, and subsequently centrifuged at 10,000 × g for 5 min. Supernatants were precipitated with cold ethanol at 4 ^∘^C for 20 min, and pellets were resuspended in distilled water. Uronic acid content was measured by reacting the samples with 12.5 mM sodium tetraborate in concentrated sulfuric acid, followed by heating at 95 ^∘^C for 5 min. Subsequently, 0.15% 3-phenylphenol prepared in 0.5% sodium hydroxide was added to initiate color formation. Absorbance at 520 nm was recorded and adjusted for bacterial concentration (CFU/mL) to determine relative CPS levels. Each assay was performed in triplicate, ensuring consistent and reliable assessment of CPS yield and uronic acid concentration in rifaximin-, berberine-, and combination-treated cultures compared to untreated controls.

### Molecular docking

Molecular docking studies were performed to evaluate the interaction of RfaH with rifaximin and berberine chloride. The RfaH structure was obtained from the AlphaFold database (ID: AF-A0A2X3CVV5-F1-model_v4). Molecular docking, ligand preparation, protein preparation, and grid preparation were conducted using InstaDock [29]. A grid box was defined with coordinates X: –4.482, Y: –1.653, Z: 0.019 and dimensions of 64 Å, 58 Å, and 78 Å for the X, Y, and Z axes, respectively. Following docking, interaction analysis was performed to identify the best-docked conformation using PyMOL [30].

### Molecular dynamics simulation

All-atom molecular dynamics (MD) simulations were conducted to evaluate the stability and efficiency of protein-ligand interactions [31, 32]. The simulations were performed using GROMACS 2022.2 [33] with the CHARMM36 [34] force field for free RfaH and RfaH-rifaximin, RfaH-berberine chloride, and RfaH-(rifaximin + berberine chloride) complexes. Ligand topologies were generated using the CGenFF web server, and explicit hydrogen atoms were added to the structures in Avogadro prior to simulation. Each protein and its protein–ligand complex were placed in a cubic simulation box with an edge length of 10 Å. The systems were solvated using the TIP3P water model, and neutralization was achieved by adding appropriate counterions. Equilibration was performed in two stages: first under constant-volume (NVT) conditions, followed by constant-pressure (NPT) conditions. The temperature was maintained at 300 K with a coupling time constant of 0.1 ps. A 200 ns production run was executed for each system. The resulting trajectories were analyzed using the built-in GROMACS tools to evaluate the dynamic behavior and overall stability of the protein–ligand complexes.

## Results

Drug repurposing, exemplified by the U.S. Food and Drug Administration (FDA) approval of favipiravir for influenza [35] and Closantel for antibiotic-resistant *Staphylococcus aureus* infections [36], presents a promising strategy to rapidly address KP infections. Building on this concept, our prior research established rifaximin, a non-systemic gut-targeted antibiotic, as a repurposed candidate against KP through its interaction with the transcriptional elongation factor RfaH. Driven by the urgent need for effective anti-KP therapies and leveraging the repurposing potential of rifaximin, this study evaluated the synergistic antibacterial activity of rifaximin combined with berberine chloride. Berberine chloride, a natural isoquinoline alkaloid with established broad-spectrum antimicrobial properties, was aimed at enhancing therapeutic efficacy against this challenging pathogen.

### Antibacterial activity of berberine chloride in combination with the antibiotic rifaximin against KP

The antibacterial efficacy of rifaximin and berberine chloride against KP ATCC 700603 was quantitatively assessed using the INT colorimetric assay to determine MIC. Individually, both agents exhibited equivalent intrinsic activity, with MICs of 100 µM. This result for rifaximin aligns with our prior investigations [18]. Notably, co-administration of the compounds using a checkerboard assay revealed a substantial synergistic enhancement of antibacterial potency. The MIC for rifaximin decreased two-fold to 50 µM, whereas the MIC of berberine chloride showed a pronounced 16-fold reduction to 6.25 µM. The checkerboard assay (Supplementary Table S1) revealed that the combination significantly reduced the effective concentration of both agents. The MIC of rifaximin decreased from 100 µM to 50 µM, while the MIC of berberine chloride decreased from 100 µM to 6.25 µM due to enhanced combinatorial activity. FICI was calculated as follows: (50/100) + (6.25/100) ═ 0.5 + 0.0625 = 0.5625. An FICI of 0.56 indicates a partially synergistic interaction.

**Table 1:** Binding constants for berberine chloride, rifaximin, and their combination

**S. No.**	**Compound name**	**Binding constant (*K*_a_) Value**
1	Berberine Chloride	1.09 × 10^6^ M^−1^
2	Rifaximin	7.38 × 10^6^ M^−1^
3	Berberine chloride + Rifaximin	6.86 × 10^7^ M^−1^

**Figure 1.**
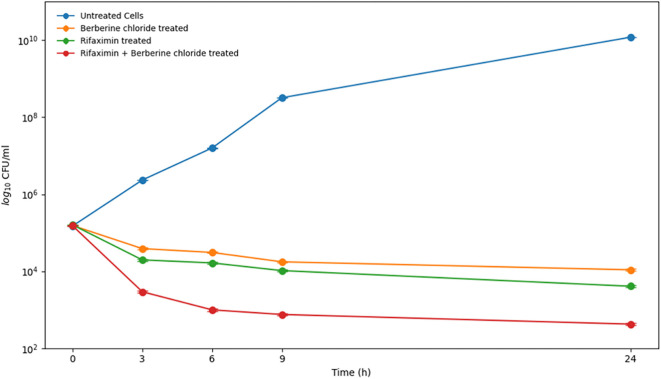
**Time–kill kinetics of KP ATCC 700603 following rifaximin and berberine chloride monotherapy or combination treatment.** Early log-phase cultures (starting inoculum ≈1.5 × 10^5^ CFU/mL) were exposed to rifaximin (100 µM), berberine chloride (100 µM), or the synergistic checkerboard-derived combination (50 µM rifaximin + 6.25 µM berberine chloride), alongside an untreated growth control, and viable counts were quantified over 24 h by colony enumeration. The combination produced enhanced killing relative to either single agent, achieving a 2.56 log_10_ CFU/mL reduction from baseline at 24 h versus 1.59 log_10_ for rifaximin alone (Δ ≈ 0.98 log_10_). This improvement did not meet the predefined time–kill synergy criterion (≥2 log_10_ CFU/mL decrease compared with the most active single agent at the same time point). Data are mean log_10_ CFU/mL ± SD from three independent experiments. Abbreviations: KP: *Klebsiella pneumoniae*; ATCC: American Type Culture Collection; CFU: Colony-forming unit; SD: Standard deviation.

Time-kill assays provided strong evidence of bactericidal synergy, defined as a ≥2 log_10_ CFU/mL decrease by the combination compared to the most effective single agent at the same time point [25]. At 24 h, the combination of 50 µM rifaximin and 6.25 µM berberine chloride achieved a 2.56 log_10_ CFU/mL reduction from the starting inoculum, surpassing the 1.59 log reduction achieved by rifaximin alone (difference ≈ 0.98 log_10_) (Figure 1, Supplementary Table S2). However, this difference did not meet the predefined threshold for time-kill synergy (≥2 log_10_ decrease compared to the most effective single agent). Time-kill assays demonstrated enhanced bactericidal activity of the combination compared to either agent alone. Collectively, these findings, encompassing MIC reduction, FICI, and dynamic kill curves, establish a compelling foundation for the therapeutic strategy of combining rifaximin with berberine chloride against multidrug-resistant KP infections.

### Synergistic enhancement of RfaH binding revealed by intrinsic fluorescence quenching

Intrinsic fluorescence spectroscopy was utilized to elucidate the molecular interactions between rifaximin, berberine chloride, and their combination with the RfaH protein. Ligand-induced changes in the local environment of aromatic residues, including tryptophan, tyrosine, and phenylalanine, were assessed through alterations in fluorescence emission.

**Figure 2.**
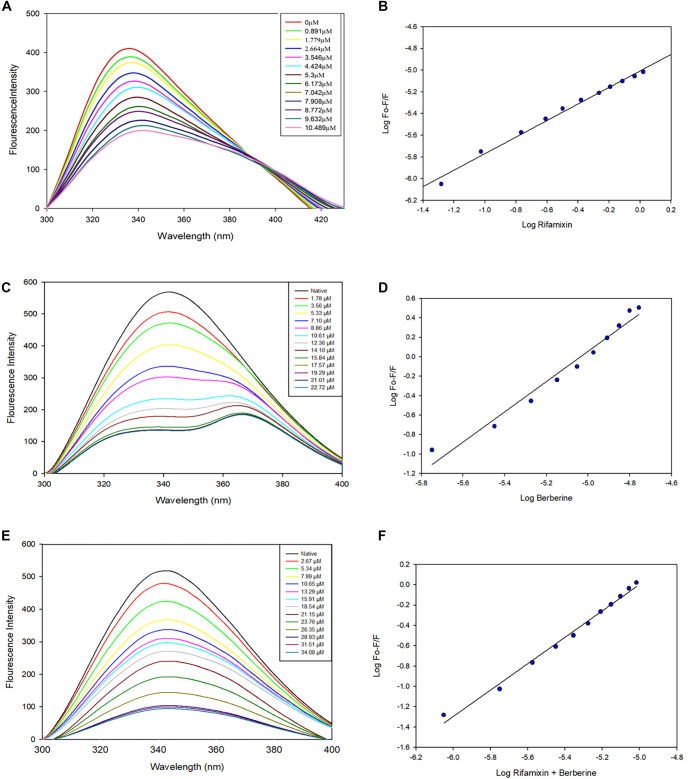
**Intrinsic fluorescence quenching of RfaH by rifaximin, berberine chloride, and their combination.** (A, C, E) Emission spectra of RfaH recorded upon titration with increasing concentrations of rifaximin, berberine chloride, or an 8:1 (rifaximin:berberine chloride) mixture. Samples were excited at 280 nm and emission was collected from 300–400 nm. Progressive quenching with increasing ligand concentration indicates direct ligand engagement with RfaH. The 8:1 mixture produced a stronger quenching response consistent with cooperative binding. (B, D, F) Modified Stern–Volmer analyses of the corresponding titrations used to estimate Ka. Rifaximin bound RfaH with high affinity (K_a_ ═ 7.38 × 10^6^ M^−^^1^), berberine chloride exhibited moderate affinity (K_a_ ═ 1.09 × 10^6^ M^−^^1^), and the 8:1 mixture yielded a markedly enhanced affinity (K_a_ ═ 6.86 × 10^7^ M^−^^1^), indicating synergistically strengthened binding. Abbreviation: RfaH: Transcriptional antitermination factor.

Titration experiments demonstrated a concentration-dependent quenching of RfaH fluorescence by both compounds, indicative of direct binding (Figure 2A and 2C). Analysis with a modified Stern-Volmer equation revealed that rifaximin binds to RfaH with high affinity (K_a_ ═ 7.38 × 10^6^ M^--1^), as established in our previous study [18]. Berberine chloride exhibited a moderate binding affinity with a K_a_ value of 1.09 × 10^6^ M^--1^ (Figure 2B and 2D). Notably, when rifaximin was combined with a reduced concentration of berberine chloride, a synergistic fluorescence quenching effect was observed (Figure 2E). This combination produced a significant quenching response, with fitting of the experimental data revealing an enhanced binding affinity of 6.86 × 10^7^ M^--1^, reflecting an almost tenfold increase over the affinities observed for rifaximin and berberine alone (Figure 2F and Table 1).

The increased K_a_ indicates cooperative binding or ligand-induced conformational optimization of RfaH when both compounds are present. The finding that halving the concentration of berberine relative to its individual efficacy within the combination not only maintained but significantly amplified RfaH engagement aligns with established fluorescence signatures of combinatorial additive molecular interactions.

### Effect of synergy on capsule production in KP

**Figure 3.**
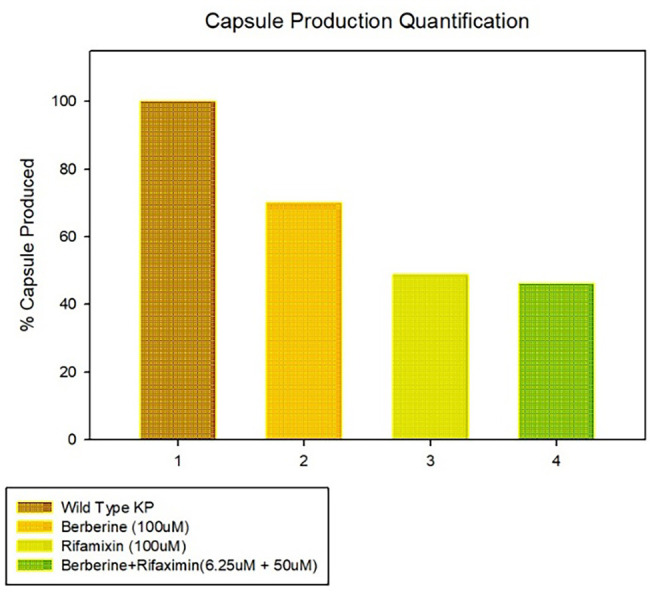
**CPS production in KP following treatment with rifaximin, berberine chloride, or rifaximin+berberine chloride.** CPS levels were quantified in KP after 16 h exposure to rifaximin (100 µM), berberine chloride (100 µM), or rifaximin+berberine chloride (50 µM + 6.25 µM). CPS is expressed as % capsule produced relative to the untreated control (set to 100%) after normalization to CFU/mL. Rifaximin alone reduced CPS by >50%, berberine chloride produced a more modest reduction (∼30%), and rifaximin+berberine chloride achieved CPS suppression comparable to rifaximin monotherapy despite a 2-fold lower rifaximin concentration. Data are representative of three independent experiments with consistent trends. Abbreviations: CPS: Capsular polysaccharide; KP: *Klebsiella pneumoniae*; CFU: Colony-forming unit.

Capsule quantification assays conducted on KP ATCC 700603 demonstrated distinct and comparative effects of rifaximin, berberine chloride, and their combination on capsule production. Treatment with rifaximin alone at 100 µM, previously shown to be effective, led to a significant reduction in capsule production of over 50% relative to the untreated control, thus confirming prior observations. The administration of berberine chloride at its inhibitory concentration resulted in a milder effect, decreasing capsule content by approximately 30%. Importantly, co-administration of rifaximin and berberine chloride at lower concentrations (50 µM and 6.25 µM, respectively) resulted in a synergistic reduction in capsule biosynthesis (Figure 3). This combination achieved a reduction comparable to that of 100 µM rifaximin alone (approximately 50%), despite utilizing only half the rifaximin concentration required to achieve this level of suppression when used as a single agent at its MIC-equivalent efficacy.

The rifaximin-berberine combination achieves synergistic capsule suppression through a dual mechanism targeting RfaH. Rifaximin directly inhibits at the functional interface, while berberine induces dysregulation via allosteric trapping. Their cooperative binding leads to a stabilized, inactive ternary complex that effectively halts the transcriptional program essential for capsule biosynthesis. This mechanistic understanding underscores RfaH as a viable target for combinatorial antimicrobial therapy and provides a rational basis for employing dual-targeting strategies to combat resilient pathogens such as KP.

### Interaction analysis of rifaximin and berberine chloride binding to RfaH

**Figure 4.**
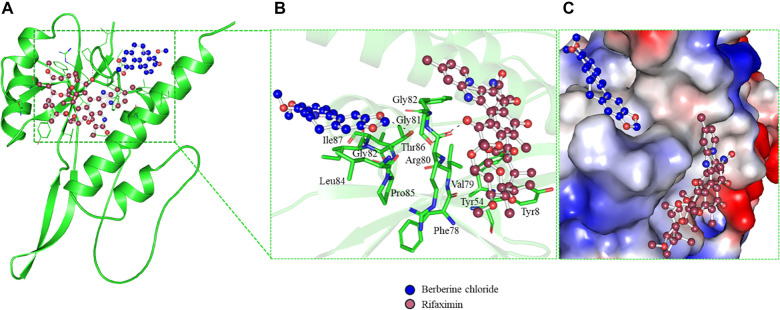
**Docking-predicted interactions of rifaximin and berberine chloride with RfaH.** (A) 2D interaction maps summarizing polar and hydrophobic contacts formed by rifaximin and berberine chloride within the RfaH binding region. (B) 3D binding poses highlighting key H-bonds with functionally relevant residues. Rifaximin engages the pocket via multiple stabilizing contacts, including an H-bond network involving Tyr54, consistent with inhibition at a critical functional interface. Berberine chloride preferentially occupies the NTD hydrophobic patch cavity and forms H-bonds with Gly81 (3.3 Å) and Thr86 (3.5 Å), a region implicated in regulating the NTD–βCTD autoinhibitory interface and post-TEC refolding; binding at this site is therefore predicted to perturb conformational switching and dysregulate RfaH activity. (C) Surface representation of RfaH illustrating pocket shape complementarity and deep ligand accommodation for both rifaximin and berberine chloride. Abbreviations: RfaH: Transcriptional antitermination factor RfaH; NTD: N-terminal domain; βCTD: Beta C-terminal domain; TEC: Transcription elongation complex; H-bonds: Hydrogen bonds; Å: ångström.

Molecular docking studies revealed that rifaximin and berberine chloride bind to the RfaH protein with calculated binding affinities of --9.3 kcal/mol and --7.3 kcal/mol, respectively. A comprehensive analysis of the nine docked conformations for each ligand was performed to characterize their interactions within the RfaH binding pocket. Rifaximin forms hydrogen bonds with critical RfaH residues Tyr54 (β′ clamp helices (CH) domain, 3.2 Å), Phe78 (3.0 Å), Arg80 (2.3 Å), and Asp147 (3.5 Å). Notably, Tyr54 serves as a universally essential contact point for RfaH function, as literature indicates that disruption of interactions at this site impairs RfaH-dependent gene activation in both *E. coli* and *KP* [28]. Therefore, rifaximin binding to Tyr54 is predicted to directly inhibit RfaH activity by interfering with this key functional interface. Rifaximin also engages in hydrophobic interactions with Tyr8 (3.93 Å), Val79 (3.73 Å), and Leu143 (3.86 Å). Visualization of the binding mode (Figure 4A–C) confirms that rifaximin binds deeply within a pocket in RfaH, interacting specifically with residues of the β′ CH domain.

Conversely, berberine chloride primarily targets the hydrophobic patch within the N-terminal domain (NTD), forming hydrogen bonds with Gly81 (3.3 Å) and Thr86 (3.5 Å). This patch is critical for stabilizing the autoinhibited α-helical state through interactions with the C-terminal domain (βCTD). Disruption of this hydrophobic surface, either by ligands or mutations, prevents the formation or maintenance of the autoinhibitory NTD-βCTD interface, rendering RfaH incapable of adopting its inactive conformation [37]. Importantly, this patch is also necessary for initiating βCTD refolding back into the α-state following RfaH dissociation from the Transcription Elongation Complex (TEC). Consequently, berberine chloride binding to this patch is predicted to hinder post-TEC refolding, effectively trapping RfaH in its active β-barrel conformation (β-state). This locked active state leads to constitutive, dysregulated RfaH activity, resulting in aberrantly high-level translation of RfaH-dependent operons (e.g., virulence factors, capsule synthesis proteins), causing toxic overexpression, energetic waste, loss of coordinated gene regulation, and compromised cellular fitness or pathogenicity due to loss of temporal control [38]. Berberine chloride binds within the NTD hydrophobic patch cavity (Figure 4A and 4B) and penetrates deeply into the RfaH pocket (Figure 4C), similarly to rifaximin.

Thus, while rifaximin inhibits RfaH by disrupting a critical functional contact point (Tyr54) within the β’CH domain, berberine chloride dysregulates RfaH by binding the NTD hydrophobic patch, preventing autoinhibition and trapping the protein in a constitutively active state. Both mechanisms exploit key interaction sites that are essential for RfaH function across bacterial species.

### Structural dynamics and stability analysis of RfaH in complex with rifaximin and berberine chloride

**Figure 5.**
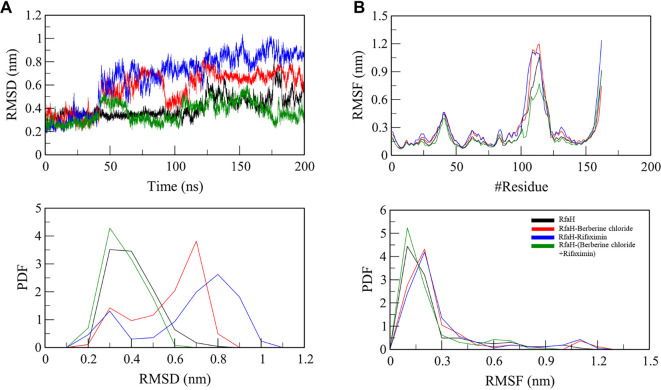
**RMSD and RMSF profiles of RfaH during 200 ns MD simulations in apo and ligand-bound states.** (A) RMSD time-evolution for apo RfaH and RfaH complexes with single ligands (rifaximin or berberine chloride) or the dual-ligand system (rifaximin+berberine chloride). Relative to apo RfaH (avg RMSD = 0.41 nm), single-ligand binding increased structural deviation (rifaximin: 0.57 nm; berberine chloride: 0.67 nm), whereas dual-ligand binding reduced RMSD (0.36 nm), indicating restoration of near-apo-like stability. The lower-left panel shows the RMSD PDF, illustrating a narrower distribution for the dual-ligand complex compared with single-ligand complexes. (B) Per-residue RMSF for apo and ligand-bound systems. Compared with apo RfaH (avg RMSF = 0.24 nm), residue-level flexibility increased with single ligands (rifaximin: 0.30 nm; berberine chloride: 0.28 nm) but decreased in the dual-ligand complex (0.21 nm), consistent with ligand-cooperative stabilization without major perturbation of the overall flexibility pattern. The lower-right panel shows the RMSF PDF for each system. Abbreviations: RMSD: Root mean square deviation; RMSF: Root mean square fluctuation; MD: Molecular dynamics; apo: Unbound; RfaH: Transcriptional antitermination factor RfaH; PDF: Probability density function.

Molecular dynamics simulations of RfaH, both in its native state and in complex with rifaximin, berberine chloride, or both ligands, provided significant insights into structural dynamics and stability through analyses of root-mean-square deviation (RMSD), radius of gyration (*R*g), solvent-accessible surface area (SASA), and root-mean-square fluctuation (RMSF). The native RfaH exhibited an average RMSD of 0.41 nm. Upon individual ligand binding, the RMSD increased to 0.57 nm with rifaximin and 0.67 nm with berberine chloride, indicating reduced stability upon binding. However, when both ligands bound simultaneously, the RMSD decreased significantly to 0.36 nm, approaching the native state. This decrease suggests that synergistic ligand binding enhances the overall structural stability of RfaH (Figure 5A).

RMSF quantifies the flexibility of individual residues in a protein by measuring the average deviation of each residue’s position over time relative to its mean position. The average for native RfaH was 0.24 nm. The rifaximin-bound complex showed an average RMSF of 0.30 nm, and the berberine chloride-bound complex displayed 0.28 nm, indicating increased residue flexibility upon individual ligand binding. In contrast, the synergistic complex exhibited a reduced average RMSF of 0.21 nm, suggesting that the combined binding of both ligands stabilizes specific residues, effectively reducing their flexibility relative to the unbound and single-ligand states (Figure 5B).

**Figure 6.**
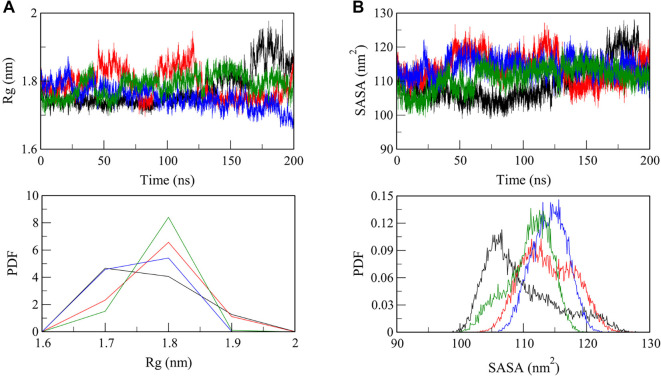
**Rg and SASA profiles of RfaH during 200 ns MD simulations in apo and ligand-bound states.** (A) Rg time-evolution for apo RfaH and RfaH complexes with rifaximin, berberine chloride, or rifaximin+berberine chloride. Mean Rg values were highly similar across systems (apo: 1.78 nm; rifaximin: 1.79 nm; berberine chloride: 1.79 nm; rifaximin+berberine chloride: 1.79 nm), indicating that ligand binding does not measurably alter global compactness. The lower-left panel shows the Rg PDF for each trajectory. (B) SASA time-evolution showing that single-ligand binding increased solvent exposure relative to apo RfaH (apo: 109.64 nm^2^; rifaximin: 114.12 nm^2^; berberine chloride: 113.92 nm^2^), whereas the dual-ligand complex shifted SASA toward the apo-like state (rifaximin+berberine chloride: 110.95 nm^2^), consistent with reduced structural perturbation under cooperative binding. The lower-right panel shows the SASA PDF for each trajectory. Abbreviations: Rg: Radius of gyration; SASA: Solvent-accessible surface area; MD: Molecular dynamics; apo: Unbound; RfaH: Transcriptional antitermination factor RfaH; PDF: Probability density function.

Analysis of compactness via the *R*g revealed minimal variation across states. The native RfaH protein exhibited an average Rg of 1.78 nm, while the ligand-bound complexes with rifaximin and berberine chloride showed *R*g of 1.79 nm. The synergistic complex also demonstrated an average *R*g of 1.79 nm. These small differences suggest that ligand binding does not significantly affect the global compactness of RfaH, maintaining the protein in a stable, compact conformation throughout the simulations (Figure 6A).

SASA analysis indicated moderate structural rearrangements. The native RfaH had a SASA of 109.64 nm^2^. Single ligand binding increased SASA (rifaximin: 114.12 nm^2^; berberine: 113.92 nm^2^). Notably, dual ligand binding reduced SASA to 110.95 nm^2^, closely resembling the native value. This similarity in SASA between the native and synergistic bound states suggests minimal structural disruption upon dual ligand binding (Figure 6B).

Hydrogen bonds are often referred to as the ‘master key of molecular interactions’ due to their ubiquity and flexibility, playing a critical role in mediating biological processes such as ligand binding and enzyme catalysis [39]. An analysis of hydrogen bonding, encompassing both intra- and intermolecular interactions, was performed to evaluate the stability of RfaH complexes. Evaluation of intramolecular hydrogen bonds within the RfaH structure over the 200 ns simulation duration revealed no significant alterations upon binding rifaximin, berberine chloride, or both ligands simultaneously, compared to the native state.

**Figure 7.**
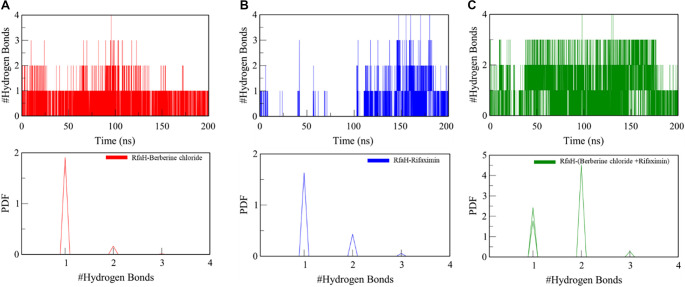
**Intermolecular H-bonding between RfaH and ligands during 200 ns MD simulations.** (A–C) Time-resolved counts of intermolecular H-bonds formed between RfaH and (A) berberine chloride, (B) rifaximin, or (C) the dual-ligand system (rifaximin+berberine chloride). Both single-ligand complexes formed up to 4 H-bonds, with an average occupancy centered at ∼1 H-bond over the trajectory; the berberine chloride complex retained ∼1 persistent H-bond. In contrast, the dual-ligand complex also reached up to 4 H-bonds but maintained ∼2 persistent H-bonds across the simulation, consistent with enhanced and more sustained intermolecular stabilization. (Lower panels) PDFs of H-bond counts show the dominant population at 1 H-bond for the single-ligand complexes, whereas the dual-ligand complex exhibits its main peak at 2 H-bonds, supporting increased H-bond occupancy under cooperative binding. Abbreviations: H-bond: Hydrogen bond; MD: Molecular dynamics; RfaH: Transcriptional antitermination factor RfaH; PDF: Probability density function.

The intermolecular hydrogen bond analysis indicated the formation of up to four hydrogen bonds for the RfaH-berberine chloride complex, with one hydrogen bond remaining consistent (Figure 7A). Similarly, the RfaH-rifaximin plots showed the formation of up to four hydrogen bonds, with an average of one (Figure 7B). In contrast, the synergistic complex also formed four hydrogen bonds, with two remaining consistent throughout the simulation (Figure 7C). The PDF plots revealed the highest peak at one for the RfaH complexes with berberine chloride and rifaximin, whereas the synergistic complex exhibited the largest peak at two, indicating that complex formation does not disrupt the protein’s internal hydrogen-bonding network.

Collective analysis of RMSD, Rg, SASA, and RMSF underscores the stabilizing effects of ligand binding on RfaH. Reduced global flexibility (lower RMSD and RMSF values), maintained compactness (Rg), and altered solvent exposure (SASA) suggest structural adjustments that improve the enzyme’s conformation for ligand binding (Table 2). These results provide valuable mechanistic insights into RfaH’s functional behavior, facilitating the rational design of effective inhibitors or modulators.

**Table 2:** Average values of structural integrity parameters for RfaH during molecular dynamics simulations in both unbound and bound states at 310 K

**System**	**RMSD (nm)**	**RMSF (nm)**	***R*g (nm)**	**SASA (nm^2^)**	**#H-bonds**
RfaH	0.41	0.24	1.78	109.64	98
RfaH-Berberine	0.57	0.28	1.79	113.92	96
RfaH-Rifaximin	0.67	0.30	1.75	114.12	98
RfaH-(Berberine+Rifaximin)	0.36	0.21	1.79	110.95	99

Abbreviations: RMSD: Root mean square deviation; RMSF: Root mean square fluctuation; Rg: Radius of gyration; SASA: Solvent-accessible surface area; H-bonds: Hydrogen bonds.

**Table 3:** Residue counts in the secondary structures of RfaH in both unbound and various bound forms at 310 K

	**RfaH**	**RfaH-berberine**	**RfaH-rifaximin**	**RfaH-(berberine+rifaximin)**
Coil	33	31	32	33
β-sheet	36	34	36	36
β-bridge	1	2	0	1
Bend	16	16	15	14
Turn	20	19	17	19
α-helix	51	53	55	53
π-helix	0	0	0	0
3_10_-helix	0	1	1	1
κ-Helix	5	6	6	5

Abbreviation: RfaH: Transcriptional antitermination factor RfaH.

### Secondary structure dynamics of native RfaH and synergistic complex

**Figure 8.**
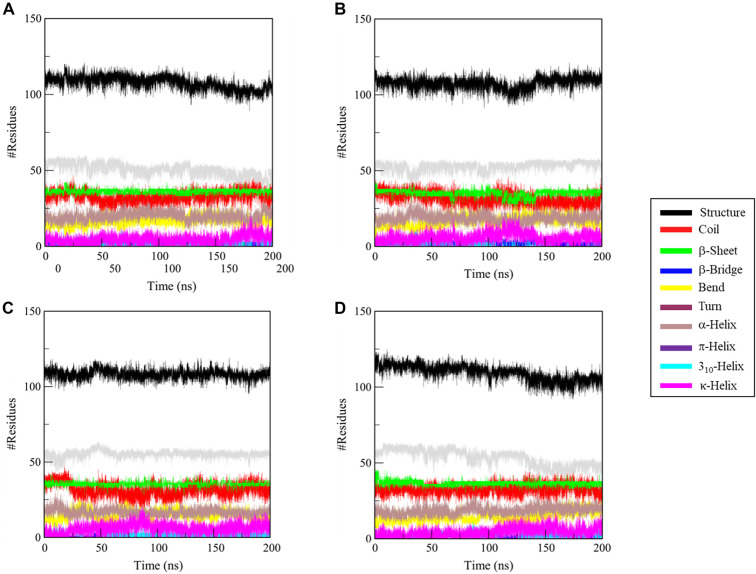
**Secondary-structure time evolution of RfaH during 200 ns MD simulations in apo and ligand-bound states.** (A–D) Secondary-structure assignments plotted as residue counts versus time for (A) apo RfaH, (B) RfaH–rifaximin, (C) RfaH–berberine chloride, and (D) RfaH–rifaximin+berberine chloride. Across all trajectories, the secondary-structure topology remained stable, with no evidence of global unfolding. The RfaH–rifaximin and RfaH–berberine chloride complexes largely preserved the apo-like α/β architecture, indicating that single-ligand binding does not destabilize the fold. The RfaH–rifaximin+berberine chloride system exhibited subtle, cooperative remodeling consistent with a minor reduction in β-structure accompanied by a modest increase in α-helical content, while retaining the overall native-like secondary-structure framework. Abbreviations: MD: Molecular dynamics; apo: Unbound; RfaH: Transcriptional antitermination factor RfaH.

Analysis of secondary structure dynamics offers critical insights into protein conformational behavior and folding stability [40]. Molecular dynamics simulations demonstrated that unbound RfaH maintains stable and equilibrated secondary structural elements throughout the 200 ns trajectory (Figure 8A). The rifaximin-bound complex also exhibited preserved secondary structure topology, indicating that binding does not induce significant unfolding or destabilization of the protein fold (Figure 8B). Upon binding to berberine chloride, RfaH displayed a similarly stable profile, with no major disruption to its core α-helical or β-sheet architectures (Figure 8C). The formation of the synergistic dual-ligand complex (RfaH-rifaximin-berberine) was accompanied by subtle conformational adjustments, characterized by a minor decrease in β-sheet content and a slight increase in α-helical structure (Figure 8B). Quantification of residues engaged in regular secondary structures (Table 3) confirmed variations across all ligand-bound states (rifaximin, berberine, and dual-ligand complex) compared to the apo protein RfaH. Crucially, despite these measurable differences, the overall secondary structure topology of RfaH remains largely unaltered upon binding rifaximin alone, demonstrating the robust structural integrity of this complex.

### Principal component analysis and free energy landscape

**Figure 9.**
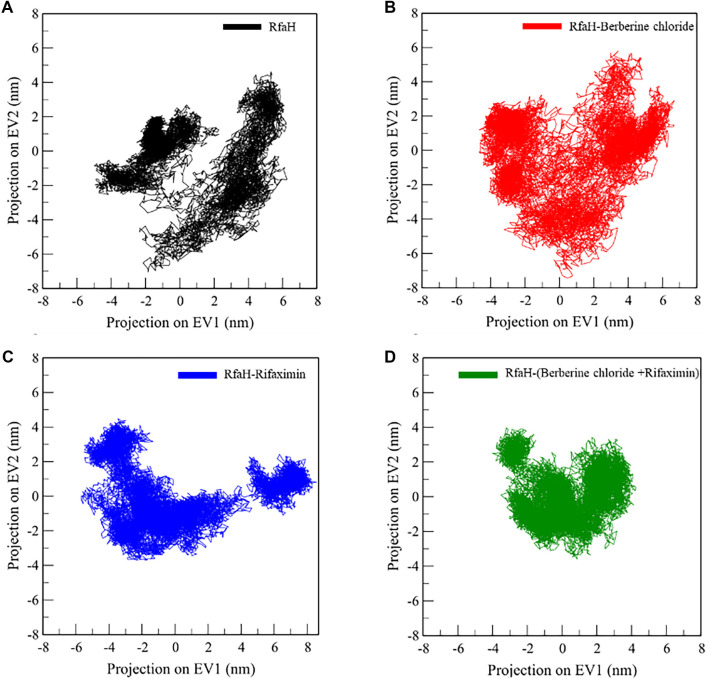
**PCA projections of RfaH conformational sampling during MD simulations.** (A–D) 2D projections of MD trajectories onto the first two principal components (EV1 and EV2), computed from Cα coordinates for (A) apo RfaH, (B) RfaH–berberine chloride, (C) RfaH–rifaximin, and (D) RfaH–rifaximin+berberine chloride. Single-ligand complexes explored conformational space comparable to, or broader than, apo RfaH, whereas the dual-ligand system occupied a markedly more confined subspace, indicating restricted collective motions and enhanced conformational stabilization of RfaH under cooperative binding. Abbreviations: PCA: Principal component analysis; MD: Molecular dynamics; EV1/EV2: Eigenvector 1/2 (principal components 1/2); Cα: Alpha carbon; apo: Unbound; RfaH: Transcriptional antitermination factor RfaH.

Principal component analysis (PCA) is a valuable computational technique for investigating protein conformational dynamics and collective atomic motions [41]. In this study, PCA was employed to examine the conformational space explored by RfaH in its unbound state and when complexed with rifaximin, berberine chloride, and the dual-ligand combination of rifaximin and berberine chloride. The analysis utilized conformational projections based on the Cα atoms of RfaH to assess structural variations before and after ligand binding (Figure 9). The results indicated that the RfaH-ligand complex occupied a more confined conformational subspace than the free protein, as well as the RfaH-rifaximin and RfaH-berberine chloride complexes. This suggests that binding the synergistic dual ligand enhances the protein’s stability while occupying its binding cavity.

**Figure 10.**
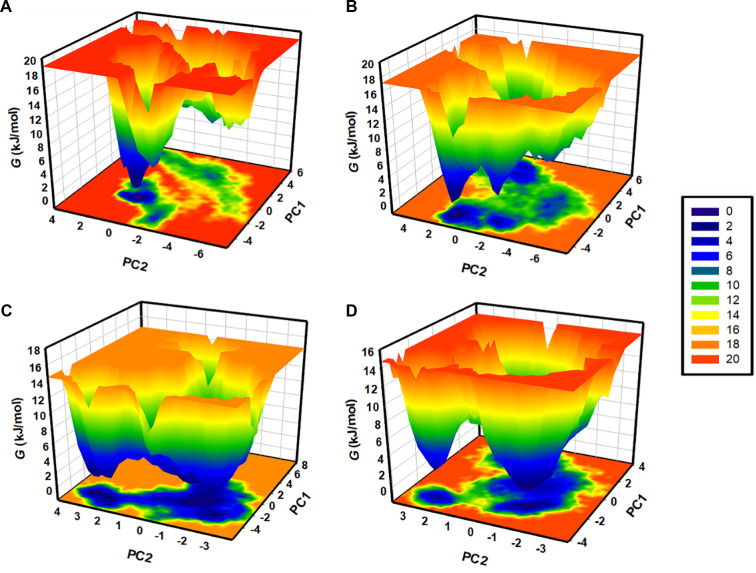
**FELs of RfaH conformational states derived from MD trajectories.** (A–D) FELs mapped onto PC1 and PC2 for (A) apo RfaH, (B) RfaH–berberine chloride, (C) RfaH–rifaximin, and (D) RfaH–rifaximin+berberine chloride. Color gradients denote relative free energy (G), with deep-blue regions representing low-energy conformations. Apo RfaH and single-ligand systems exhibit multiple, dispersed minima, consistent with broader conformational sampling and heterogeneous low-energy states. In contrast, the dual-ligand system displays a dominant, deep-blue basin with a secondary smaller basin, indicating a more focused low-energy ensemble and increased conformational stabilization of RfaH under cooperative binding. Abbreviations: FEL: Free energy landscape; MD: Molecular dynamics; PC1/PC2: Principal component 1/2; G: Gibbs free energy; apo: Unbound; RfaH: Transcriptional antitermination factor RfaH.

Free energy landscapes (FELs) are crucial for analyzing protein folding kinetics and thermodynamic parameters. Utilizing the MD simulation trajectories, FELs provide insights into the conformational space and solvation of protein-ligand complexes. For RfaH, RfaH-rifaximin, RfaH-berberine chloride, and RfaH-(rifaximin and berberine chloride), we generated FELs to determine energy minima and conformational maps (Figure 10). The shaded deep-blue areas on the FELs correspond to low-energy conformations close to the native state. The contour maps of FELs for these complexes reveal that the binding of rifaximin and berberine chloride alters the minimum energy regions of the RfaH protein. In the RfaH complex with the dual-ligand system, there is a single large, deep blue basin and a smaller basin, whereas in the other three systems, the basins are scattered. This indicates stable binding of the synergistic complex with the RfaH protein.

## Discussion

Bacterial infections pose a growing global health threat due to antimicrobial resistance and the decline of antibiotic development pipelines, leading to increased morbidity and mortality from untreatable infections [42, 43]. Natural compounds, such as isoquinoline alkaloids, exhibit broad-spectrum antimicrobial activity and are widely distributed in nature [44]. These compounds represent promising candidates against multidrug-resistant pathogens and have undergone extensive investigation [45]. However, the rapid evolutionary capacity of bacteria necessitates the discovery of novel strategies to prevent the development of resistance. While numerous potent natural antimicrobials have entered clinical trials, their deployment carries the inherent risk of adaptive resistance that can negate efficacy. A key strategy to mitigate the risk of clinical resistance involves utilizing natural compounds in synergistic antimicrobial combinations [46]. Such synergistic interactions enhance lethality, potentially lowering the probability of bacterial escape mutants and subsequent resistance evolution. This combinatorial approach focuses on identifying pairs that yield effective antimicrobial synergy to prolong the utility of these compounds.

Our study demonstrates enhanced combinatorial activity between berberine chloride and rifaximin against KP. While the FICI (0.56) indicates partial synergy in static MIC assays, while time-kill data revealed an additive to moderately enhanced bactericidal effect, with the combination achieving approximately 0.98 log_10_ greater killing than rifaximin alone at 24 h—a meaningful enhancement, albeit below the predefined ≥2 log_10_ threshold for time-kill synergy. Building on our prior study showing that rifaximin targets the essential anti-terminator factor RfaH in KP, molecular docking reveals that both rifaximin and berberine chloride occupy the RfaH binding pocket and form hydrogen bonds with functionally critical residues. This dual binding is supported by intrinsic fluorescence quenching assays, where a 1:1 mixture shows additive quenching of RfaH and a tenfold increase in the association constant (K_a_) relative to single agents, indicating enhanced binding affinity.

RfaH transcriptionally regulates capsule production in KP by acting as an anti-terminator at the ops site within the promoter-distal region of the cps (capsular polysaccharide) operon, enabling full-length expression of genes essential for capsule biosynthesis [47]. When evaluating the repurposing potential of an existing drug, two critical parameters are enhanced efficacy and improved specificity in the new therapeutic context. The rifaximin-berberine combination demonstrates significant advantages in both areas compared to rifaximin monotherapy. While rifaximin alone shows moderate activity, its partnership with berberine chloride dramatically enhances potency, reducing the MIC of berberine by 16-fold to 6.25 µM and that of rifaximin by 2-fold to 50 µM. This indicates that the combination can achieve robust therapeutic outcomes at lower, potentially safer doses of the repurposed drug. Regarding specificity, the combinatory actions through RfaH inhibition offer a targeted anti-virulence strategy rather than a broad-spectrum bactericidal approach.

Rifaximin, in its original indication, acts locally in the gut with minimal systemic absorption. Its repurposed mechanism, as revealed here, is highly specific and directly binds to and inhibits a master regulator of virulence. By targeting RfaH, the combination specifically disarms the pathogen by suppressing capsule production, a key virulence determinant, without exerting the strong selective pressure for resistance typically associated with essential target inhibition. This dual improvement highlights a highly favorable profile for drug repurposing. This strategy offers a pragmatic and powerful approach to rapidly deploy refined, combination-based therapy against multidrug-resistant KP, potentially extending the lifespan and utility of both an existing drug and a natural antimicrobial compound.

While this study presents a compelling case for RfaH-targeted synergy, its limitations must be acknowledged. The findings, derived from *in vitro* models using a single KP strain, require validation across diverse clinical isolates to confirm broad applicability. Furthermore, the exclusive focus on RfaH inhibition leaves unexplored the plausible contribution of off-target effects to the observed synergy.

It is important to note that the experimental work in this study was performed using *Klebsiella quasipneumoniae* subsp. *similipneumoniae* (ATCC 700603), a strain frequently employed as a reference in antimicrobial resistance studies. While this strain shares core genetic and pathogenic mechanisms with clinically prevalent KP isolates, particularly the conserved role of the RfaH regulator in capsule biosynthesis and virulence, extrapolating our synergistic efficacy data to diverse clinical KP strains, especially hypervirulent or carbapenem-resistant variants, requires further validation. This represents a limitation of the present *in vitro* study. Future work will prioritize evaluating the berberine-rifaximin combination against a panel of contemporary, multidrug-resistant KP clinical isolates to confirm the broad applicability of this combinatorial efficacy and its potential impact on antimicrobial resistance in a clinically relevant context.

Additionally, we must prioritize *in vivo* validation in animal models and expand testing to diverse clinical isolates. Mechanistic studies should delineate off-target effects using transcriptomics and rigorously assess the combination’s ability to suppress resistance emergence. For clinical translation, developing novel formulations to overcome rifamycin’s poor systemic absorption is crucial. Ultimately, this work establishes a platform for discovering next-generation RfaH-targeted anti-virulence therapies against multidrug-resistant pathogens.

## Conclusion

This study demonstrates that berberine chloride and rifaximin act synergistically against KP. Integrated *in silico* and biophysical data support a model in which this synergy involves dual targeting of the transcriptional anti-terminator RfaH. Mechanistically, both compounds concurrently occupy the binding pocket, forming stabilizing interactions with key residues, as evidenced by molecular docking, intrinsic fluorescence quenching, and molecular dynamics simulations confirming ternary complex stability. Functionally, the combination achieved a 2.56 log_10_ reduction in bacterial viability from the starting inoculum and suppressed capsule production at half the rifaximin concentration required for monotherapy. These findings validate RfaH as a druggable target and support the development of combinatorial strategies to enhance antimicrobial efficacy while reducing effective doses. The repurposing of rifaximin with berberine offers a promising clinical strategy against KP by leveraging their synergistic, bactericidal action via dual RfaH inhibition. This combination could enhance treatment efficacy against multidrug-resistant strains while potentially reducing the development of resistance. The main therapeutic advantage lies in its anti-virulence mechanism, which disrupts capsule production without necessitating intense selective pressure. Clinically, the established safety profile of rifaximin facilitates rapid repurposing, and this combination could become a novel, targeted therapy for resistant KP infections.

## Supplemental data

**Table S1:** Results of the checkerboard assay for rifaximin and berberine chloride against KP ATCC 700603

**Rifaximin (µM) ↓ Berberine (µM) →**	**100**	**50**	**25**	**12.5**	**6.25**	**3.125**	**1.56**	**0.78**
**100**	--	--	--	--	--	+	+	+
**50**	--	--	--	--	**MIC** **Combo**	+	+	+
**25**	--	--	--	+	+	+	+	+
**12.5**	--	--	+	+	+	+	+	+
**6.25**	--	+	+	+	+	+	+	+
**3.125**	+	+	+	+	+	+	+	+

Note: Growth: +; No Growth: --. Abbreviation: MIC: Minimum inhibitory concentration.

**Table S2:** Time-kill kinetics of rifaximin, berberine chloride, and their combination against KP (atcc 700603)

**Time (Hr)**	**Replicate**	**Control**	**Berberine (100 µM)**	**Rifaximin (100 µM)**	**Combination (50 µM Rif + 6.25 µM Ber)**
**0.0**	1	5.176	5.188	5.204	5.182
	2	5.172	5.182	5.199	5.178
	3	5.180	5.193	5.210	5.186
**Mean ± SD**	**5.176 ± 0.004**	**5.188 ± 0.006**	**5.204 ± 0.005**	**5.182 ± 0.004**	
**3.0**	1	6.369	4.588	4.295	3.467
	2	6.364	4.562	4.267	3.439
	3	6.375	4.612	4.320	3.493
**Mean ± SD**	**6.369 ± 0.006**	**4.587 ± 0.025**	**4.294 ± 0.026**	**3.466 ± 0.027**	
**6.0**	1	7.201	4.489	4.216	2.997
	2	7.196	4.470	4.190	2.964
	3	7.207	4.506	4.241	3.028
**Mean ± SD**	**7.201 ± 0.006**	**4.488 ± 0.018**	**4.216 ± 0.026**	**2.996 ± 0.032**	
**9.0**	1	8.507	4.245	4.017	2.877
	2	8.502	4.225	3.991	2.851
	3	8.511	4.263	4.040	2.901
**Mean ± SD**	**8.507 ± 0.005**	**4.244 ± 0.019**	**4.016 ± 0.025**	**2.876 ± 0.025**	
**24.0**	1	10.079	4.037	3.610	2.626
	2	10.072	4.009	3.586	2.600
	3	10.086	4.064	3.633	2.651
**Mean ± SD**	**10.079 ± 0.007**	**4.036 ± 0.028**	**3.610 ± 0.024**	**2.626 ± 0.026**	

Note: Data are presented as mean ± standard deviation (SD) from three independent experiments (*n* ═ 3). The term “Rif+Ber” refers to the combination of rifaximin (50 µM) and berberine chloride (6.25 µM). The starting inoculum was approximately 5.18 log_10_ CFU/mL. The results indicate that rifaximin resulted in a 1.594 log_10_ reduction in viable counts (5.204 – 3.610), while the combination treatment achieved a 2.556 log_10_ reduction (5.182 – 2.626). Abbreviations: KP: *Klebsiella pneumoniae*; ATCC: American Type Culture Collection; Rif: Rifaximin; Ber: Berberine chloride; SD: Standard deviation.

## Data Availability

The data supporting the findings are available within the article and supplementary materials.
